# Safety culture of multidisciplinary teams from neonatal intensive care units of public hospitals*

**DOI:** 10.1590/1518-8345.2849.3167

**Published:** 2019-08-19

**Authors:** Karine Antunes Marques Notaro, Allana dos Reis Corrêa, Andréia Tomazoni, Patrícia Kuerten Rocha, Bruna Figueiredo Manzo

**Affiliations:** 1 Fundação Hospitalar do Estado de Minas Gerais, Maternidade Odete Valadares, Belo Horizonte, MG, Brasil.; 2 Universidade Federal de Minas Gerais, Escola de Enfermagem, Belo Horizonte, MG, Brasil.; 3 Universidade Federal de Santa Catarina, Departamento de Enfermagem, Florianópolis, SC, Brasil.

**Keywords:** Patient Care Team, Patient Safety, Organizational Culture, Intensive Care Units, Neonatal, Neonatology, Health Personnel, Equipe de Assistência ao Paciente, Segurança do Paciente, Cultura Organizacional, Unidades de Terapia Intensiva Neonatal, Neonatologia, Pessoal de Saúde, Grupo de Atención al Paciente, Seguridad del Paciente, Cultura Organizacional, Unidades de Terapia Intensiva Neonatal, Neonatología, Personal de Salud

## Abstract

**Objective:**

analyze the safety culture of multidisciplinary teams from three neonatal intensive care units of public hospitals in Minas Gerais, Brazil.

**Method:**

a cross-sectional survey conducted with 514 health professionals, using the Hospital Survey on Patient Safety Culture; data were subjected to a descriptive statistical analysis in software R-3.3.2.

**Results:**

the findings showed that none of the dimensions had a positive response score above 75% to be considered as a strength area. The dimension ‘Nonpunitive response to error’ was classified as a critical area of the patient safety culture, present in 55.45% of the responses. However, areas with potential for improvements were identified, such as ‘Teamwork within units’ (59.44%) and ‘Supervisor/manager’s expectations and actions to promote patient safety’ (49.90%).

**Conclusion:**

none of the dimensions was considered as a strength area, which indicates safety culture has not been fully implemented in the evaluated units. A critical look at the weaknesses of the patient safety process is recommended in order to seek strategies for the adoption of a positive safety culture to benefit patients, family members and health professionals.

## Abstract (pt)

**Objetivo:**

analisar a cultura de segurança da equipe multiprofissional em três Unidades de Terapia Intensiva Neonatal de hospitais públicos de Minas Gerais, Brasil.

**Método:**

estudo tipo *survey*, transversal, desenvolvido com 514 profissionais de saúde. Utilizou-se o instrumento Hospital Survey on Patient Safety Culture, sendo os dados submetidos à análise estatística descritiva por meio do *software* R, versão 3.3.2.

**Resultados:**

os achados mostraram que nenhuma dimensão obteve escore de respostas positivas acima de 75% para ser considerada como área de força. A dimensão “Resposta não punitiva ao erro” foi classificada como área crítica da cultura de segurança do paciente com 55,45% das repostas. Contudo, foram identificadas áreas com potencial para melhoria, como “Trabalho em equipe na unidade” (59,44%) e “Expectativas e ações do supervisor/chefia para a promoção da segurança do paciente” (49,90%).

**Conclusão:**

nenhuma das dimensões foi considerada como área de força, o que aponta que a cultura de segurança ainda não está integralmente implementada nas unidades avaliadas. Recomenda-se um olhar crítico sobre as fragilidades do processo de segurança dos pacientes, a fim de buscar estratégias para a adoção de uma cultura de segurança positiva, beneficiando pacientes, familiares e profissionais.

## Abstract (es)

**Objetivo:**

analizar la cultura de seguridad del equipo multiprofesional en tres Unidades de Cuidados Intensivos Neonatales en hospitales públicos de Minas Gerais, Brasil.

**Método:**

estudio tipo *survey*, transversal, desarrollado con 514 profesionales de salud. Se utilizó el instrumento Hospital Survey on Patient Safety Culture, y los datos se sometieron a análisis estadístico descriptivo por medio del *software* R, versión 3.3.2.

**Resultados:**

los resultados revelaron que ninguna dimensión obtuvo puntuación de respuestas positivas por encima del 75% para ser considerada como área de fuerza. La dimensión «Respuesta no punitiva al error» fue clasificada como área crítica de la cultura de seguridad del paciente con el 55,45% de las repuestas. Sin embargo, se identificaron áreas con potencial de mejora, como «Trabajo en equipo en la unidad» (59,44%) y «Expectativas y acciones del supervisor/jefe para la promoción de la seguridad del paciente» (49,90%).

**Conclusión:**

ninguna de las dimensiones se consideró como área de fuerza, lo que indica que la cultura de seguridad aún no está integralmente implementada en las unidades valoradas. Se recomienda una mirada crítica sobre las fragilidades del proceso de seguridad de los pacientes, con el fin de buscar estrategias para la adopción de una cultura de seguridad positiva, para beneficiar a pacientes, familiares y profesionales.

## Introduction

Patient safety is one of the critical pillars of health care quality and discussions about it have been strengthened after the publication of the American report *To err is human: building a safer health system*, which highlights the great number of errors and damages involved in health care^(1)^.

After that, studies on safety culture assessment and impact on health management have been considered crucial for the development of safe care, with emphasis on learning, continuous improvement and nonpunitive response to error^(2)^. Safety culture is characterized as the product of individual and collective values, attitudes, skills and behavior patterns, which determine the commitment, style and proficiency of a healthy and safe organization^(3)^.

Safety culture in health care settings is usually assessed through quantitative questionnaires based on individual items and a combination of dimensions^(2-4)^. One study reports that institutions with a positive safety culture offer safe and better quality of care to their patients. In addition, better rates in safety culture assessments may help reduce occurrences of infection and adverse events^(4)^.

Patient safety can be influenced by the work culture of the multidisciplinary team involved. A study reports that many elements of work culture directly affect health care, especially due to the way health professionals see patient safety and perform their work^(5)^.

In settings such as neonatal intensive care units (NICUs), where patients are more vulnerable and the daily routine of the multidisciplinary team involves many error-prone processes^(6)^, analyzing the safety culture becomes critical to identify areas with potential for improvements.

Then, studies that measure the safety culture in institutions are becoming an essential component of safety management systems. Some initiatives have been reported, but few studies have been conducted in neonatology focused on the safety culture of multidisciplinary teams.

According to the findings in the area, evaluating the safety culture allows the development of dimensions related to patient safety in the context of NICUs, which may imply planning actions for safer and better quality of neonatal care.

Considering evaluation as a critical action in the search of safety culture, this study aimed to analyze the safety culture of multidisciplinary teams from three NICUs of public hospitals in Minas Gerais, Brazil.

## Method

This is a cross-sectional quantitative survey conducted in three NICUs of large public hospitals in Belo Horizonte, reference institutions for high-risk pregnancy in the State of Minas Gerais. The three study sites were named A, B and C, and they have similar characteristics, such as the occupancy rate of 90% to 100%; the patient safety center; and the children presenting different levels of complexity and the main diagnosis of premature birth, leading to longer periods in the NICU; and the fact that most health professionals from the multidisciplinary team are hired through civil service exams. Due to their similar profile, the units were not analyzed by scenario.

This study used intentional and non-probability sampling, and the inclusion criteria were: the health professional had to be a physician, nurse, nursing technician, speech therapist, physical therapist, occupational therapist, social worker or psychologist, providing direct care to patients and/or accompanying people, and performing their duties in the units during the period of data collection. The exclusion criteria were: professionals who had been working in the unit for less than three months, which was considered the minimum period for adaptation to the unit; professionals on vacation or away from work; professionals who failed to return the questionnaire or who returned it with more than 50% incomplete responses. Based on these criteria, 734 professionals from the three units were selected to participate in the study. Of this total, 194 were excluded and 36 were lost due to incomplete responses and failed to return the questionnaire, totaling 514 participants from three NICUs: 211 from unit A, 130 from unit B, and 173 from unit C.

Despite using intentional and non-probability sampling, this study analyzed sample representativeness considering the three NICUs are reference units in the State of Minas Gerais. This analysis was performed using the method for estimating proportions for finite populations, with proportional allocation to title/role. Considering a 6% margin of error and a 5% level of significance, the sample calculation that ensures sample representativeness would be at least 130 professionals from unit A, 110 professionals from unit B, and 140 professionals from unit C. The sample was representative in all units, with all of them presenting the minimum sample size required, stratified by title/role.

Before starting this study, an authorization was obtained from the authors responsible for the translation and validation of the Hospital Survey on Patient Safety Culture (HSOPSC) instrument, of the Agency for Healthcare Research and Quality (AHRQ), validated for the Brazilian hospital context^(7)^. Data collection was performed between November 2016 and February 2017, through verbal and individual presentation of the study project to the professionals from multidisciplinary teams. Then, an informed consent form (ICF) and an envelope containing the instrument of data collection were delivered to participants. Data collection instrument was filled individually by the study participants during working hours, then it was placed in a box in the unit for anonymity.

The HSOPSC has nine sections, with a total of 42 items distributed in 12 areas or dimensions of patient safety culture, and three levels: I) work area/unit (supervisor/manager’s expectations and actions to promote patient safety, organizational learning – continuous improvement, teamwork within units, communication openness, feedback and communication about errors, nonpunitive response to errors, and adequacy of human resources); II) hospital organization (management support to patient safety, teamwork across units, handoffs and transitions); and III) result (overall perceptions of patient safety and frequency of events reported). The two result questions (patient safety score and number of adverse events reported in the last 12 months) are assessed separately, without constituting a dimension^(7)^.

The primary endpoint was the proportion of positive responses in each domain of the HSOPSC. Demographic variables (sex, age, educational level) and professional variables (professional category, time of work in the institution, weekly hours) were collected for sample characterization.

Responses to the instrument were coded using a five-point Likert scale (agreement: I strongly disagree, I disagree, I do not agree or disagree, I agree, I strongly agree; frequency: never, almost never, sometimes, almost always, always). The results were evaluated considering the performance of each item and dimension^(7)^.

For descriptive analysis, the responses were recoded, noting that not all items of the 12 dimensions were answered in the instruments, causing a difference in total responses of each dimension. The proportion of responses in each item was calculated, and reliability of the domains was calculated using Cronbach’s alpha. Values of >0.5 were considered of good reliability.

The responses provided in each dimension were classified in areas of strength or critical areas^(7-8)^. Areas of strength were those presenting 75% of ‘strongly agree/agree’ or ‘almost always/always’ responses to positively worded questions, and ‘strongly disagree/disagree’ or ‘never/almost never’ for negatively worded questions. Critical areas were those presenting 50% or more participants answering negatively with ‘strongly disagree/disagree’ or ‘never/almost never’ for positively worded questions, and ‘strongly agree/agree’ or ‘almost always/always’ for negatively worded questions^(7-8)^.

The presentation of results considered the following distribution by professional category: physician, nurse, nursing technician, and others (social worker, speech therapist, physical therapist, psychology and occupational therapy). The category ‘others’ gathers all other professionals due to the reduced number of participants.

Data was analyzed in software R-3.3.2, and the indicators were compared to categorical variables using Mann-Whitney and Kruskal-Wallis statistical tests. In addition, when the Kruskal-Wallis test showed a significant difference, the Nemenyi test was used for multiple comparisons.

This project was based on Resolution 466/2012 of the National Health Council (CNS) and Operational Directive 001 of 2013 of the CNS. It was submitted with the ICF to the Research Ethics Committee of the Federal University of Minas Gerais (UFMG) for approval – protocol 1.862.502.

## Results

This study had the participation of 514 health professionals, corresponding to 70% of the professionals working in all three NICUs during data collection period. Of these, most were female, 472 (91.8%), predominant age group 31 to 40 years old (n=279; 54.3%), followed by 21 to 30 years old (n=97; 18.87%), time of work in the institution: 1 to 5 years (n=251; 49.7%); and 20 to 39 weekly hours (n=243; 48.1%). Table 1 shows other characteristics of study participants.

**Figure 1:**
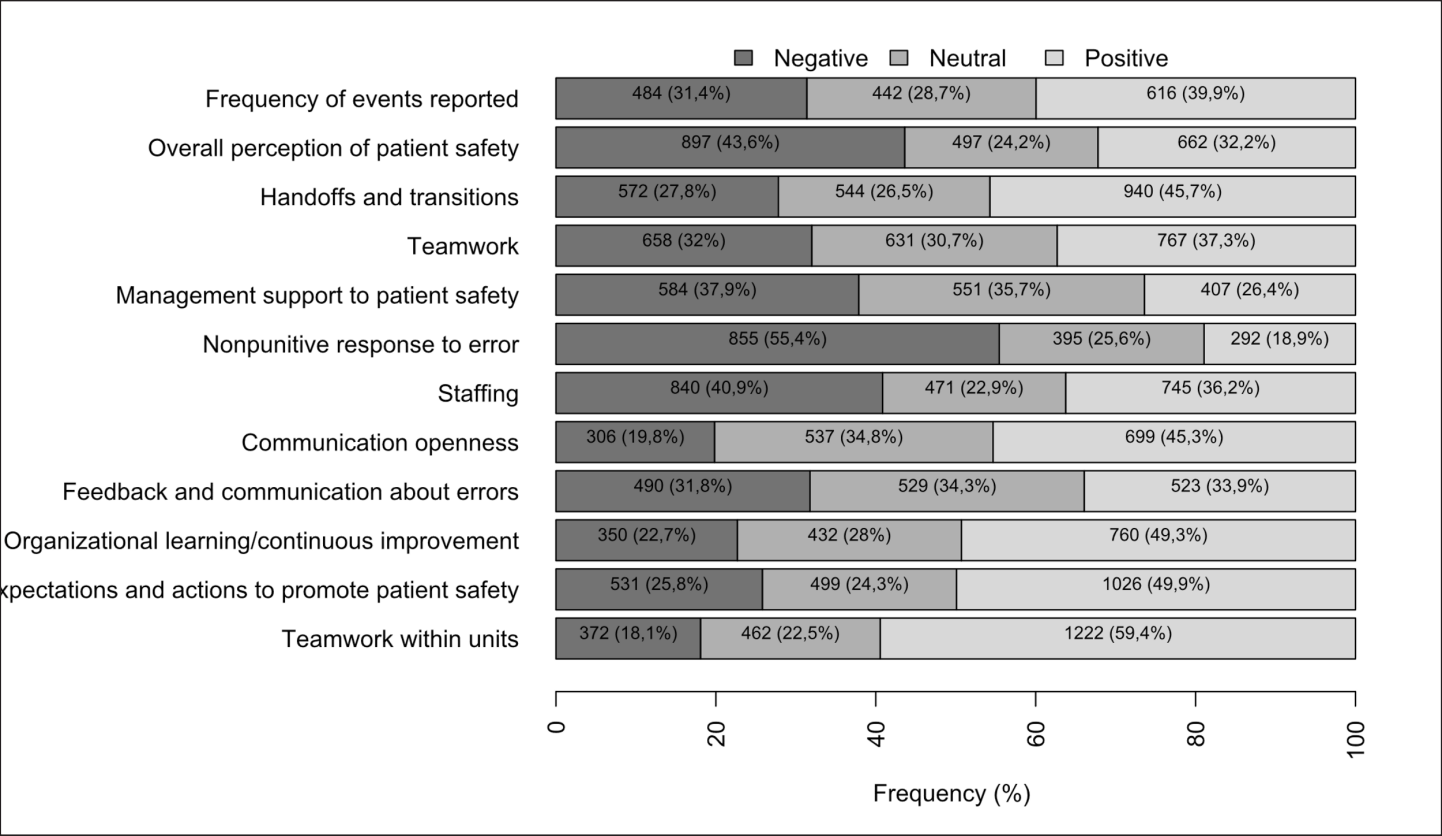
*HSOPSC – Hospital Survey on Patient Safety Culture*.* – Distribution of positive, neutral and negative responses provided to 12 dimensions of the patient safety culture, according to the HSOPSC* in the three neonatal intensive care units. Belo Horizonte, MG, Brazil, 2017

All 42 items related to patient safety in the HSOPSC provided 721 (39.5%) positive responses, 578 (32.2%) negative responses, and 458 (28.1%) neutral responses. The items were grouped, creating 12 dimensions. This way, means of positive, negative and neutral responses were obtained for each dimension, as illustrated in Figure 1.

**Table 1:** – Characteristics of health professionals who work in three neonatal intensive care units. Belo Horizonte, MG, Brazil, 2017 (n=514)

Variables		n (%)
Sex	Female	472 (91.8)
	Male	42(8.2)
Professional category	Nursing technicians	223(43.4)
	Nurses	121(23.5)
	Physicians	79(15.4)
	Other*	91(17.7)
Time of work in the institution (years)^†^	Less than ‘ year	48(9.5)
	1 to 5 years	251(49.7)
	6 to 10 years	106(20.9)
	11 to 15 years	48(9.6)
	16 to 20 years	24(4.7)
	21 years or more	28(5.6)
Weekly hours^†^	Less than 20 hours a week	(1.8)
	20 to 39 hours a week	243(48.1)
	40 to 59 hours a week	226(44.7)
	60 to 79 hours a week	27(5.4)
Educational level^†^	Elementary education	3(0.6)
	High school	191(37.8)
	Higher education	66(13.1)
	Postgraduate (specialization course)	219(43.4)
	Postgraduate (master’s or doctor’s degree)	26(5.1)

*Other: speech therapists, physical therapists, occupational therapists, social workers and psychologists; †Variables with nine interviews, missing information.

According to the guidelines from the Agency for Health Research and Quality, data obtained in this study did not show any dimension with a positive response score above 75% to be considered a strength area. That is, of the 12 dimensions evaluated, 11 were characterized as weakness or opportunities for improvement, and none as a strength area. However, some dimensions presenting a higher percentage of positive responses and the items of these dimensions received a better evaluation^(8)^.

Then, the dimension of ‘Teamwork within units’, characterized by support and respect among employees and teamwork, represented 1,222 (59.4%) positive responses, which is the highest percentage of all dimensions. This dimension has four items; of these, the item with the best evaluation was “In this unit, staff treat each other with respect’ as 349 (67.9%) of all participants agreed with this statement.

The dimension with the second highest percentage of positive responses was ‘Supervisor/manager’s expectations and actions to promote patient safety,’ which is characterized by supervisor/manager’s attitudes to promote safety. In this context, this dimension represented 1,026 (49.9%) positive responses. This dimension has four items as well; of these, the best evaluated item was ‘My supervisor/boss does not give enough attention to recurring patient safety problems,’ as 325 (63.2%) participants disagreed with this statement, with a positive impact on safety culture.

The third most important dimension was ‘Organizational learning – continuous improvement,’ which refers to a learning culture where errors are analyzed, leading to positive changes. This dimension obtained 760 (49.3%) positive responses. Of the three items that constitute this dimension, the most relevant item was ‘We are actively doing things to improve patient safety,’ with 335 (65.2%) participants agreeing with this statement.

On the other hand, some dimensions presented a high percentage of negative responses. Those dimensions with a score of 50% or higher were classified as critical areas of the patient safety culture. The critical area with the highest percentage was ‘Nonpunitive response to error,’ that is, when errors are not used in a punitive manner. This dimension obtained 855 (55.4%) negative responses. It has three items, and those representing the biggest obstacles were ‘Professionals consider that their errors can be used against them,’ with 308 (59.9%) responses, followed by ‘Professionals are concerned about their errors being recorded in their employment history,’ with 294 (57.2%) responses.

The second dimension classified as potential critical area was ‘Overall perception of patient safety,’ which considers procedures and systems are adequate to avoid errors, mistakes or failures and do not present patient safety problems. This dimension obtained 897 (43.6%) negative responses, and most participants (n=331, 64.4%) disagreed with the item ‘Patient safety is never compromised due to the greater amount of work to be performed’ and 267 (51.9%) of them agreed that ‘More serious errors do not happen here by chance.’

‘Staffing’ was the third dimension with potential for becoming a critical area in the study, as it assumes proper number of staff to handle the workload. This dimension obtained 840 (40.8%) negative responses, and 340 (66.1%) perceived problems in the item ‘We have enough professionals to deal with the amount of work,’ suggesting the number of professionals is not enough.

**Figure 2:**
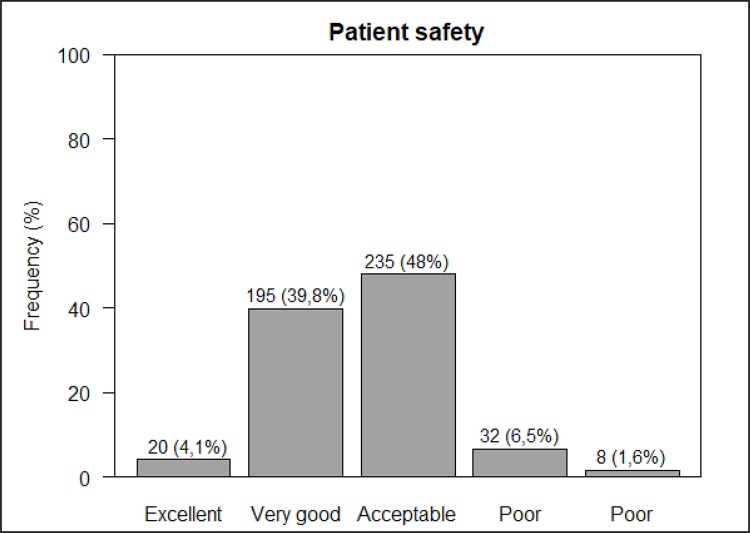
– Distribution of responses about patient safety score in three neonatal intensive care units of public hospitals. Belo Horizonte, MG, Brazil, 2017

In addition to assessing the dimensions of safety culture, the instrument of data collection presented two variables of safety culture results. The first one refers to the professional’s perception of patient safety, with a safety score provided for his/her unit. Data showed that almost half of the participants evaluated safety culture as ‘acceptable’ and 39.8% considered safety as ‘very good’ (Figure 2).

**Figure 3:**
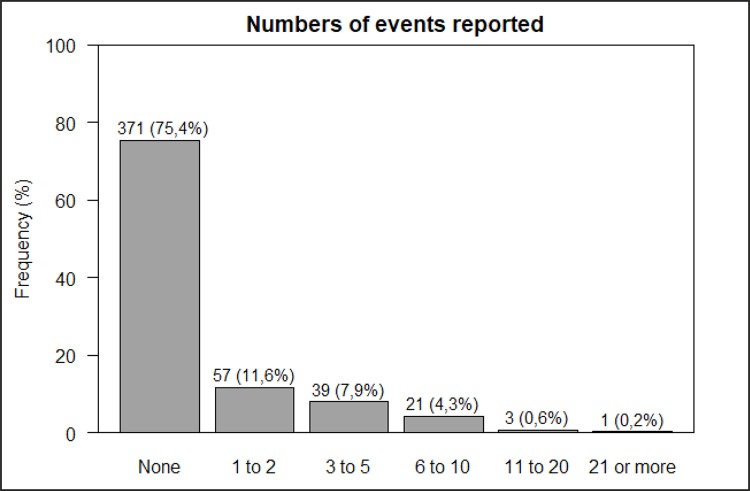
– Distribution of the number of events reported to the supervisor/manager in the last 12 months in three neonatal intensive care units of public hospitals. Belo Horizonte, MG, Brazil, 2017

The second result variable of safety culture shows the number of events reported by the health professional to his/her supervisor/manager in the last 12 months. Most (75.4%) of the respondents did not report events during this period (Figure 3).

The authors of this study decided to evaluate the distribution of responses of result variables for safety culture according to the professional category. A significant difference (p=0.005) was observed among the titles, with 8% of nursing technicians classifying safety as ‘excellent,’ while for physicians, nurses and other professionals, the proportions were 1.4%, 0.8%, and 1.1%, respectively (Table 2).

**Table 2:** – Comparison of result variables: patient safety assessment and adverse events reported, according to the professional categories in three neonatal intensive care units of public hospitals. Belo Horizonte, MG, Brazil, 2017

Variables		Physician		Nurse		Technician		Other*		p value^†^
		n	%	n	%	n	%	n	%	
Patient safety	Excellent	1	1.4	1	0.8	17	8	1	1.1	0.005
	Very good	34	45.9	41	35.3	92	43.2	28	32.2	
	Acceptable	35	47.3	64	55.3	87	40.8	49	56.3	
	Poor	4	5.4	9	7.8	11	5.2	8	9.2	
	Very poor	-	-	1	0.8	6	2.8	1	1.2	
Events reported (forms filled and sent to supervisor/manager)	No event reported	66	83.6	45	38.2	183	88.4	77	87.5	<0,001
	1 to 2 events reported	11	13.9	25	21.2	15	7.3	6	6.8	
	3 to 5 events reported	2	2.5	28	23.7	7	3.4	2	2.3	
	6 to 10 events reported	-	-	16	13.6	2	0.9	3	3.4	
	11 to 20 events reported	-	-	3	2.5	-	-	-	-	
	21 or more events reported	-	-	1	0.8	-	-	-	-	

*Other: Speech therapists, physical therapists, occupational therapists, social workers, and psychologists; †p value <0.05.

Regarding the number of events reported (forms filled and sent to supervisor/manager), a significant difference (p<0.001) was observed, and no event was reported by 38.2% nurses, 83.6% physicians, 88.4% nursing technicians, and 87.5% among the other categories. Events were mostly reported by nurses (61.8%), followed by nursing technicians (19.7%), physicians (16.4%) and the other categories considered in the study (12.5%).

## Discussion

The results show that safety culture is not fully established in the NICUs, which is similar to other studies^(6,9)^. However, some dimensions presented the highest percentage of positive responses, but also below 75%, among them, the dimensions of ‘Teamwork within units’ and ‘Supervisor/manager’s expectations and actions to promote patient safety.’ International studies have reported similar findings^(10-11)^, as well as Brazilian studies^(12)^. Despite regional cultural specificities, the percentage of positive responses to ‘Supervisor/manager’s expectations and actions to promote patient safety’ and ‘Teamwork within units’ obtained in these sites are similar to the percentage obtained in this study.

Regarding the dimension of ‘Teamwork within unit,’ based on the responses provided, the professionals of the units perceive respect and support among them. Regarding the dimension of ‘Supervisor/manager’s expectations and actions to promote patient safety,’ the respondents highlighted involvement and actions of supervisor/manager in the units. Of note, such involvement and actions of leaders are crucial to favor safe care by encouraging the health team to learn lessons from reported errors.

In contrast, some dimensions were classified as critical areas of a patient safety culture. Regarding the dimension of ‘Nonpunitive response to error,’ in an American study conducted in 653 general hospitals with 405,281 health professionals using the HSOPSC instrument, the dimension of ‘Nonpunitive response to error’ received 56% negative response and was the dimension with the worst evaluation^(9)^. It is evident a culpability culture blames an individual for an error, discouraging him/her to report the error and, consequently, prevents organizational learning from such occurrence^(13)^.

Regarding the dimension of ‘General perception of patient safety,’ the item with the worst evaluation was ‘Patient safety is never compromised due to the greater amount of work to be performed,’ probably due to the professional’s perception of the daily workload in the unit and the insufficient staff to meet the demand of care provision. A study with a multidisciplinary team from eight public hospitals in the region of Murcia, Spain^(10)^, showed similar results to this study, with a high percentage of negative responses in this dimension.

Despite not showing strength areas for patient safety, but critical areas only, most professionals classified patient safety as ‘acceptable’ and ‘very good.’ A study conducted in a public general hospital in Minas Gerais, Brazil, also found similar assessment of patient safety, ranging from ‘acceptable’ (43%) to ‘very good’ (40%)^(14)^.

Regarding events reported, most professionals responded they had not filled out any event form. This situation is even more alarming when a consensus is observed among experts in the subject stating the reported numbers of adverse events are a very modest estimate versus the actual number^(1)^. In addition, the number of adverse events reported should not be the responsibility of a single professional category, as found in this study. The responsibility for safety should be equally shared by all teams.

Also, a safety culture may be perceived differently according to the professional category. Nursing technicians and physicians considered patient safety as ‘very good.’ Nursing technicians were also the ones who provided ‘excellent’ responses more often. The categories of ‘nurse’ and ‘other’ were the professionals with the highest percentages of ‘acceptable’ and ‘poor’ responses. A study in the South region of Brazil with professionals from an intensive care unit (ICU) team reported that, regarding an assessment of patient safety level, most health professionals (51.93%) – and 61.12% of physicians and physical therapists – considered the ICU patient safety level as acceptable. On the other hand, most nurses considered patient safety in the ICU as poor^(11)^. In contrast, a study conducted in a chain of public hospitals in the region of Murcia, Spain, showed that nurses were more positive about safety assessment than physicians^(15)^.

Based on the knowledge in the literature regarding the role of nurses, these professionals who are trained to be critical and responsible for managing the team, the investigators have concluded that the categories of ‘nursing technician’ and ‘physician’ overestimated the evaluation because they are not aware of the process safety in its entirety. Physicians and nursing technicians are often left out of the analysis of indicators and management of event reports, which may have influenced the findings. Then, physicians and nursing technicians should be incorporated into the discussion about safety, since they would feel as part of this process and participate more actively in safety improvements in the unit.

Just like the category of nurses, the category of ‘other professionals’ also obtained the highest percentages in ‘acceptable’ and ‘poor’ scores for patient safety assessment, which indicates other professionals of the multidisciplinary team, as they are less numerous in the team, are responsible for assistance and quality management. Therefore, they are expected to be more familiar with safety culture indicators.

Regarding the variable of ‘Events reported in a form,’ the category of nurses reached the highest percentage of event reports when compared to physicians, technicians and other professionals. A study conducted in a NICU with the nursing and medical staff found that nursing technicians presented the lowest number of event reports. Nurses and physicians had 11 to 20 reported events (80% and 20%, respectively)^(5)^. Sometimes, a nurse has the responsibility to report events, as he/she is considered the most capable leader to manage adverse reports of events and encourage the team^(16)^. A study reports the need to encourage the communication of events by the multidisciplinary team in order to collectively develop strategies for error prevention and promotion of a consolidated safety culture^(16)^. The authors emphasize that, despite the fact that error reporting is a responsibility of the whole team, the hospital management needs to assume a leadership position, encouraging and implementing a safety culture that addresses errors in a systemic and non-punitive manner^(6)^.

The lack of reports from technicians/assistants and physicians was probably due to ‘corporatism,’ fear or lack of knowledge of error reporting systems, and due to the perception that incident reporting may not result in improvements^(17)^. In relation to the category of ‘other professionals,’ except for physical therapists, the other professionals (psychologist, occupational therapist, social worker and speech therapists) present a lower risk of adverse events when compared to other professionals; therefore, they report less frequency when compared to other categories. Then, further studies using this approach are suggested.

By encouraging a safety culture with continuous vigilance and monitoring processes, considering error as an opportunity for organizational learning, a continuous cycle of action and reflection is developed, enabling hospitals to learn from their experiences and create and promote an ability to reflect on the dynamics of the system, driving changes in the perception of patient safety. On the other hand, the persistent use of traditional and quick solutions to solve existing problems may inhibit more effective forms of organizational learning^(18-19)^.

This way, studies suggest that senior management commitment to support the development of a patient safety culture, the use of information technology and simulators to reduce errors, incentive to error reporting practices, and educational practices are essential for enhancing a safety culture^(19-20)^.

Regarding the insertion of ‘patient safety’ in the organizational environment and, consequently, in the organizational culture, it should be noted that it is influenced by the labor and power relations existing among the various professional profiles that constitute a hospital environment^(18)^. Then, in order to establish a patient safety culture across several professional categories, the managers in charge should lead this multidisciplinary team and promote a work environment based on dialogue and learning. Another aspect to be considered is that event reporting should be incorporated into the daily routine of these professionals, establishing a culture of learning. A national study emphasizes the need for institutional investments in the promotion and development of safe health systems^(12)^.

A limitation of this study referred to the patient culture assessment performed in public hospitals only, not including private hospitals. Then, safety culture in NICUs should be further explored in more details to formulate strategies and ensure safety.

However, the findings of this study come from interviews with 70% of all eligible employees from three NICUs, using a validated instrument to measure safety culture; they probably represent other contexts of public hospitals. This study used a multidisciplinary team approach, allowing different perspectives of the safety culture and preventing a specific view of an NICU professional and data bias.

With the recent public policies specifically related to the study theme, culture should be investigated as an opportunity to support patient safety strategies, encouraging error reporting. Through communication of errors and non-punitive culture, it is possible to identify issues and implement barriers to reduce situations of risk in health units.

## Conclusion

The findings of this study did not present any of the dimensions assessed regarding a patient safety culture as a strength area, although the professionals presented opportunities for improvement. The dimension of ‘Teamwork within units’ dimension presented the highest percentage of responses among all dimensions, followed by ‘Supervisor/manager’s expectations and actions to promote patient safety’ and ‘Organizational learning – continuous improvement.’

Data showed that almost half of the participants rated the safety culture as acceptable. When compared to the distribution of responses according to the professional category, a percentage of nursing technicians classified it as excellent, unlike other categories. In addition, it showed that, regardless of the profession, the participants presented resistance to error reporting.

Then, the investigation and discussion of the dimensions that involve a safety culture, through the application of the HSOPSC instrument, can contribute to improvements in the work process of professionals inserted in an ICU, especially the neonatal ICU. This team experiences stressful and unexpected situations on a daily basis when providing care to patients, with a high degree of vulnerability. Therefore, a critical look at patient safety process failures is recommended in order to identify gaps that need to be addressed to allow a stronger and positive safety culture to benefit patients, family members and professionals. A mature systemic vision is required to build and enhance a safety culture in health care settings.
